# Prevalence of Deletional Alpha Thalassemia and Sickle Gene in a Tribal Dominated Malaria Endemic Area of Eastern India

**DOI:** 10.1155/2014/745245

**Published:** 2014-03-11

**Authors:** Prasanta Purohit, Snehadhini Dehury, Siris Patel, Dilip Kumar Patel

**Affiliations:** ^1^Sickle Cell Clinic & Molecular Biology Laboratory, Veer Surendra Sai Medical College, Burla, Sambalpur, Odisha 768017, India; ^2^Department Medicine, Sickle Cell Clinic & Molecular Biology Laboratory, Veer Surendra Sai Medical College, Burla, Sambalpur, Odisha 768017, India; ^3^Department of Medicine, Sickle Cell Clinic & Molecular Biology Laboratory, Veer Surendra Sai Medical College, Qr. 3R/27, Doctors Colony, Burla, Sambalpur, Odisha 768017, India

## Abstract

Inherited hemoglobin disorders like alpha thalassemia and sickle gene are common in the Indian subcontinent. These disorders in the heterozygous state act as malaria resistance genes and influence the susceptibility to *Plasmodium falciparum* malaria. There is inadequate knowledge about the epidemiology of these malaria resistance genes in the tribal dominated malaria endemic region of the state of Odisha in eastern India. A cross sectional prevalence study was undertaken in 594 subjects in five tribal populations in this region, namely, Sahara (42.4%), Kutia Kandha (30.0%), Kuda (15.8%), Gond (9.8%), and Oraon (2.0%). Sickling test, Hb electrophoresis, HPLC, and molecular studies were undertaken to diagnose the prevalence of sickle allele, **β**-thalassemia allele, and deletional alpha thalassemia. Sickle and **β** thalassemia alleles were found in 13.1% and 3.4% of subjects, respectively. Sickle allele was found both in heterozygous (10.1%) and homozygous state (3.03%). The prevalence of alpha thalassemia was 50.84% with an allelic frequency of 0.37. Both **α** ^−3.7^ and **α** ^−4.2^ alpha thalassemia were detected with an allele frequency of 0.33 and 0.04, respectively. The high prevalence of alpha thalassemia and sickle gene in this population is probably due to selection pressure of endemic malaria in this part of India.

## 1. Introduction

Inherited hemoglobin disorders are the commonest monogenic diseases in humans [1]. Between 3,00,000 and 4,00,000 babies are born each year with serious hemoglobin disorders and up to 90% of these births occur in low and middle income countries [2]. Many of these inherited hemoglobin disorders are malaria resistance genes and in the heterozygous state influence the susceptibility to* P. falciparum* malaria [3, 4]. However our knowledge regarding their epidemiology and the pathophysiological basis of malaria protection is inadequate [3].

Alpha thalassemia, a commonly encountered inherited hemoglobin disorder presents in one of four clinical phenotypes (a) clinically asymptomatic (occurring in silent carrier state) due to a single alpha gene deletion (–*α*/*αα*) with no or little hematological changes [5], (b) alpha thalassemia minor due to deletion of two genes (–*α*/–*α*; – –/*αα*), (c) hemoglobin H disease due to deletion of three of the four alpha genes (– –/–*α*), and (d) Barts hydrops fetalis, a fatal hemoglobin produced due to deletion of all four alpha genes (– –/– –) [5]. Both alpha thalassemia minor and hemoglobin H lead to a phenotype resembling *β* thalassemia intermedia [4]. Alpha thalassemia is especially frequent in Mediterranean countries, South East Asia, Africa, and the Indian subcontinent [1]. It most frequently results in a mild form of alpha thalassemia due to single gene deletion from the chromosome-16 [6]. Although alpha thalassemia has been documented from various parts of India [7–12], there is no population based cross sectional prevalence study of this disorder from the state of Odisha.

The sickle gene, another malaria resistance gene is widely distributed in various parts of the world including the Indian subcontinent, where carrier frequencies range from 5% to 40% [4]. The sickle gene is highly prevalent in western districts of Odisha with a frequency of 21% [13]. In a few tribes studied, the frequency of sickle gene was found to be 8% [14].

Tribal persons are considered to be the indigenous people of the land and they usually live in isolates in natural and unpolluted surroundings away from civilization. The Indian subcontinent comprises 635 different communities including 75 primitive tribes. The state of Odisha located in eastern India occupies a unique place in the tribal map of the country with the largest number of tribal communities (62 tribes including 13 primitive tribes) [15]. Due to lack of confounding factors like migration and population admixture, this population is ideally suited for studying the relationship of alpha thalassemia, sickle gene, and historical malaria endemicity [16].

Malaria is an important public health problem in India and contributes two-thirds of the parasitologically confirmed malaria cases in the Southeast Asia region [17]. The state of Odisha located in eastern India contributes to 23% of malaria positive cases, 50% of* P. falciparum* cases, and 15% of the deaths in India [18]. Majority of the tribal population in the state of Odisha live in high risk areas of malaria [19].

Accurate assessment of the prevalence of various hemoglobin disorders like alpha thalassemia and sickle cell anemia is important for two reasons. (1) Improvement in hygiene, nutrition, and the control of infection has led to reduced childhood mortality and increased survival of babies with severe hemoglobin disorders like sickle cell anemia and thalassemia and presents a challenge for limited health resources in developing countries like India. As a result of such demographic changes, the impact of these diseases is now being felt all over the Indian subcontinent [4]. In this context, reliable estimates of the populations affected by hemoglobin disorders are desirable to guide public-health priority settings [20]. (2) Emerging resistance of anopheles mosquitos against conventional insecticides and drug resistance strains of* P. falciparum* has led to global resurgence of* P. falciparum* malaria. Host resistance genes like alpha thalassemia and sickle gene influence the population structure of* P. falciparum*, notably in the genes of* P. falciparum* that affects the success and virulence in infection [21]. Studies of these malaria protection genes in the human host and their interaction with* P. falciparum* will advance our understanding of malaria pathogenesis leading to development of an effective malaria vaccine and improved treatment strategies. The existing knowledge about the epidemiology of malaria resistance genes like alpha thalassemia and sickle gene in the tribal dominated malaria endemic region of Odisha is inadequate, because of which we undertook this study.

## 2. Materials and Methods

The study was undertaken at Sickle Cell Clinic and Molecular Biology Laboratory, Veer Surendra Sai Medical College, Burla, located in the state of Odisha, in eastern India. This hospital caters for a population of about 40 million from the western part of Odisha state and adjoining districts of Chhattisgarh state.

This cross sectional prevalence study was carried out in three tribal dominated villages situated in western Odisha with a population of 403, 394, and 432, respectively. Two of the villages were in the district of Bargarh and the other in Kalahandi district. In the three villages 48.6% (196/403), 49.2% (194/394), and 47.2% (204/432) of the population were sampled. When combined 48.3% (594/1229) of the total population in the three villages was studied. It was undertaken as a component of Odisha Sickle Cell Project, under which field studies are carried out in western districts of Odisha to estimate the prevalence of inherited hemoglobin disorders. During the field studies, a routine health check-up was carried out on all the subjects. Detailed personal information and clinical characteristics were entered in a predesigned format. Information about the importance of malaria, alpha thalassemia, and sickle cell anemia and their inheritance pattern were imparted to the villagers including various screening methods and importance of vaccination of children. The villagers were made aware of the facilities for free diagnosis, treatment, and counselling of the inherited hemoglobin disorders at the nodal centre of Veer Surendra Sai Medical College, Burla.

Written informed consent was obtained from the study subjects or guardians (in case of children) before collecting the blood by trained personnel, which is a part of ethical clearance from the institute. Five milliliters of EDTA venous blood was collected in vacutainer (Becton, Dickinson and Company) and stored between 2 and 8 degrees in a vaccine carrier and transported to the Sickle Cell Clinic and Molecular Biology Laboratory within 24 hours for various hematological and molecular analyses.

Sickling slide test and alkaline agarose gel hemoglobin electrophoresis (pH-8.6) were carried out within 3 days. All samples were subjected to Cation-exchange high performance liquid chromatography (CE-HPLC) analysis using Variant II-*β* thalassemia short programme (Bio-Rad laboratories, Hercules, CA, USA) to determine the quantities of different hemoglobin variants. Genomic DNA was extracted from whole blood by a standard phenol chloroform extraction method [22]. The presence of the *β* ^S^ mutation was confirmed by amplified refractory mutation system-PCR (ARMS-PCR) [23]. Subjects with HbA_2_ of more than 3.5% were analyzed for *β* thalassemia mutations (*β* ^T^) by procedures described earlier [24]. Alpha globin gene deletions (*α* ^−3.7^ and *α* ^−4.2^) were detected by gap-PCR [25].

The study was approved by the Institutional Ethical Committee.

## 3. Results

In the present study 594 subjects were included, of which 390 (65.7%) belonged to the district of Bargarh and the rest 204 (34.3%) were from Kalahandi district. The subjects belonged to five tribal communities namely Sahara (252, 42.4%), Kandha (Kutia), (178, 30.0%), Kuda (Mirdhas) (94, 15.8%), Gond (58, 9.8%), and Oraon (12, 2.0%). Majority of the Sahara were found in the district of Bargarh (94.4%). Kandha and Oraon were found in Kalahandi district and Gond and Kuda in Bargarh district. The mean age of the study subjects was 21.5 ± 18.7 years (range, 1–80 years) and 50.8% were female (Table 1).

**Figure 1:**
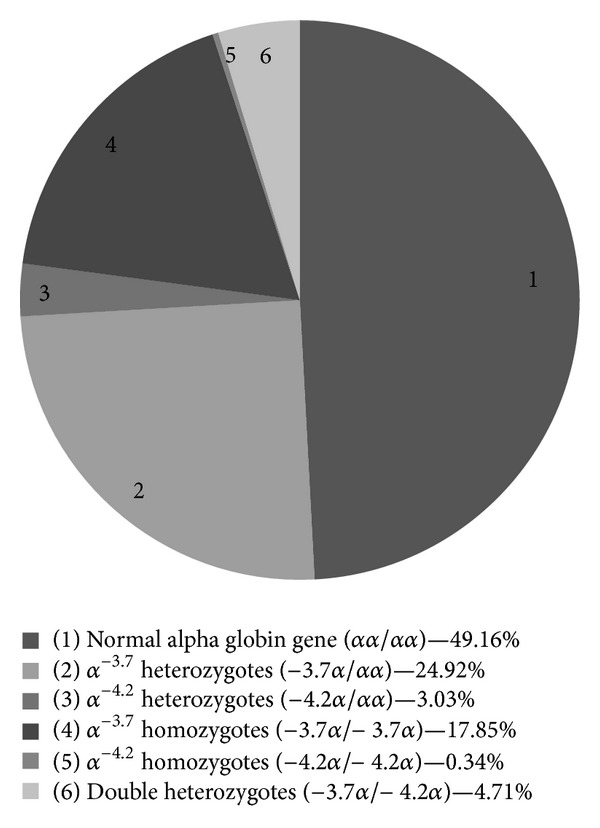
Prevalence of deletional alpha thalassemia alleles in the study subjects (*n* = 594).

The prevalence of alpha thalassemia alleles in this study population was 50.84% (Figure 1) with an allelic frequency of 0.37. Both *α* ^−3.7^ and *α* ^−4.2^ deletional alpha thalassemia were detected with an allelic frequency of 0.33 and 0.04, respectively (Table 1). The details of alpha thalassemia distribution are depicted in Table 1 and Figure 1.

**Figure 2:**
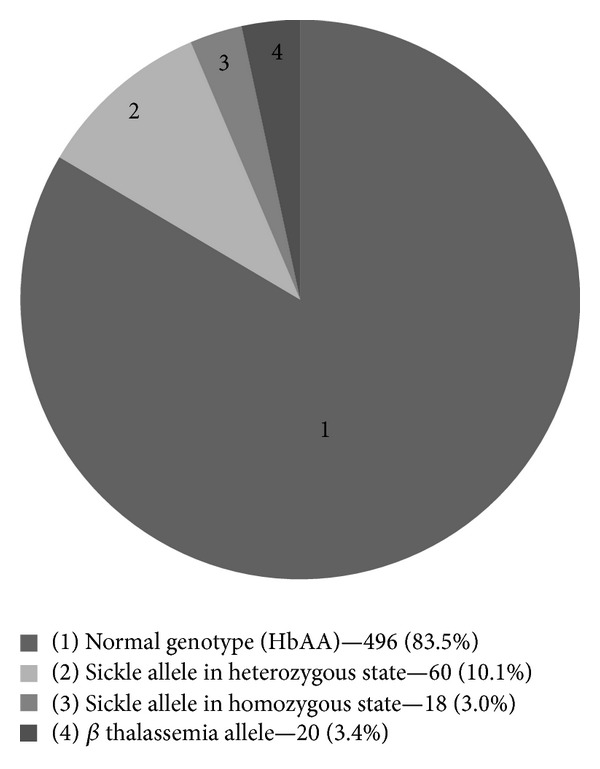
Prevalence of sickle allele and *β* thalassemia allele in the study subjects (*n* = 594).

**Figure 3:**
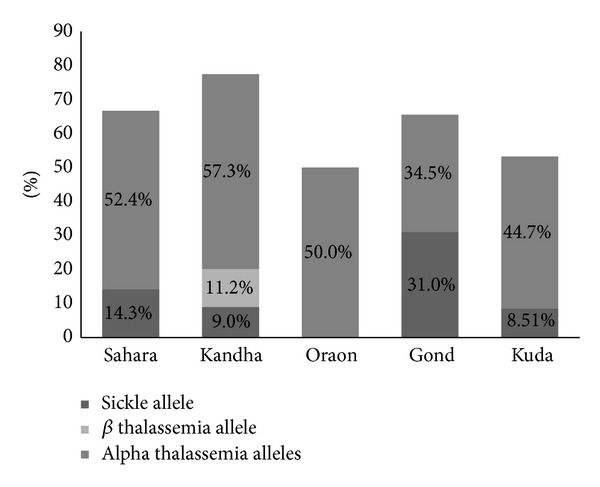
Prevalence of alpha thalassemia allele, sickle allele, and *β* thalassemia allele in five tribal communities in study subjects.

The prevalence of sickle allele was 13.1% with an allelic frequency of 0.08. Sickle allele was found both in heterozygous state (10.1%) and homozygous state, that is, sickle cell anemia (3.03%) (Figure 2). The highest prevalence of sickle allele was observed in Gond (31.0%) followed by Sahara (14.3%), Kandha (9%), and Kuda (8.51%). We did not find sickle allele in Oraons (Figure 3). Heterozygous *β* thalassemia was found in 3.37% of subjects and allelic frequency was 0.017 (Table 1). None of the subjects had beta thalassemia major or compound heterozygous state of sickle-*β* ^+^ thalassemia. IVS1-5 (G→C) was the only *β* thalassemia mutation detected and all subjects belonged to Kandha tribe. In Khandha tribe all the three alleles (sickle allele, *β* thalassemia, and alpha thalassemia) were found. Sahara, Gond, and Kuda had two alleles (sickle allele and alpha thalassemia) and the Oraon had only one allele, that is, alpha thalassemia.

The prevalence of sickle allele, alpha thalassemia allele and *β* thalassemia allele were compared in four age groups (1–15 years, >15–30 years, >30–45 years and >45 years) of the subjects (Table 2).

## 4. Discussion

Various evolutionary forces such as natural selection against malaria and social behaviors like endogamy are the most likely contributing factors for increased prevalence of inherited hemoglobin disorders in India [11]. However there is lack of well-designed epidemiological and molecular studies to confirm the malaria hypothesis for the high prevalence of these genetic disorders. Moreover accurate estimation of these hemoglobin disorders would be desirable for proper utilization of health resources in a developing country like India.

In the state of Odisha, tribes constitute 22.1% of the total population and they are 9.7% of the total tribal population of India [15]. More than 60% of these tribal populations live in malaria endemic areas. Various epidemiological studies and malariometric surveys carried out in this population revealed high frequency of* P. falciparum* malaria, as malaria control in such settlements is inadequate due to technical and operational problems [19]. This is the first community study to estimate the frequency of deletional alpha thalassemia (*α* ^−3.7^ and *α* ^−4.2^) and sickle gene and *β* thalassemia in the tribal dominated state of Odisha in eastern India.

In the Indian subcontinent the prevalence of alpha thalassemia alleles varies from 10 to 25%, although in a few localized communities they are found in 80% of the population [26]. The prevalence of alpha thalassemia in the tribal population of the state of West Bengal and Assam in eastern India has been studied in detail. In Santals of West Bengal the prevalence was 80% [8, 9], whereas in Assam the frequency in the Garos and Ahoms tribe was 36.3% and 11.62%, respectively [9]. Population survey carried out from nine ethnic groups residing in or near the Nilgiri hills in southern India revealed interesting findings. Sickle cell trait was found in 27% of population and these heterozygotes showed a trimodal distribution of the HbS concentration postulating the presence of genotypes with two (–*α*/–*α*), three (–*α*/*αα*), and four (*αα*/*αα*) active *α* globin gene. The concentration of HbS was low (27%) and these subjects had microcytic and hypochromic red cells indicating high prevalence of alpha thalassemia. Although alpha thalassemia was not confirmed by any molecular method, the authors hypothesized that high prevalence of both alpha thalassemia and sickle cell trait in these populations could be due to selection pressure exerted by hyperendemic* P. falciparum* malaria in this region [27].

There is no cross sectional community based study on the prevalence of alpha thalassemia in the tribal population of western Odisha. In an earlier hospital based study conducted on sickle cell disease patients attending to Veer Surendra Sai Medical College, Burla, the prevalence of alpha thalassemia was found to be 54.5% with an allelic frequency of 0.29 [7]. In the present study, the prevalence of alpha thalassemia was highest (56.2%) in Kandha and lowest in Gond (34.5%). *α* ^−3.7^ deletion was ubiquitous in all the tribes whereas *α* ^−4.2^ was observed in Kandha, Kuda, and Sahara tribes. Malaria is the foremost public health problem in this part of Odisha [19]. 15% of all admissions in the Department of Internal Medicine are due to severe malaria especially in the rainy season of June to September. Recently we undertook a study on the influence of alpha thalassemia on the severity of* P. falciparum* malaria in this region. We observed that alpha thalassemia had a protective effect against severe* P. falciparum *malaria (Supplementary table in Supplementary Materials available online at http://dx.doi.org/10.1155/2014/745245).

In the Indian subcontinent the prevalence of sickle gene is found to be 5–40% with an allelic frequency of 0.031–0.41 [28]. The western part of Odisha lies in the high prevalence zone. The prevalence of sickle gene in the tribal population as reported from various Indian states is 0–24% in Maharashtra [29], 24% in Chhattisgarh [30], 6.9–18.65% in Southern Gujarat [31], and 9.2% in Rajasthan [32]. In a hospital based study, the prevalence of sickle allele has been reported to be 8.2% in the tribal population of western Odisha [14]. In this study the prevalence of sickle allele was 13.1% with an allelic frequency of 0.08. Sickle gene was found in four out of the five tribes studied. The prevalence was highest (31.0%) in Gond with an allelic frequency of 0.17. Sahara tribe constituted the majority of the population (42.4%) and the prevalence of sickle allele was 14.3% in them which is higher than the earlier report of Kar [14]. Kandha was the largest tribe in the district of Kalahandi. The prevalence of sickle allele was found to be 9.0% in Kandha of Odisha state which is similar to earlier report by Sharma et al. [33]. For the first time we studied the Kuda (Mirdhas) tribe of western Odisha and the prevalence of sickle allele was lowest (8.51%) with an allelic frequency of 0.05. Sickle allele was not found in Oraon tribe. Similar finding has been reported by Kaur et al. [34]. The lack of sickle allele in the Oraon tribe could be due to recent migration of the Oraon population with founder effect. In this study this population constituted of only 2% of the total subjects studied and were localized to one village.

We compared the prevalence of sickle allele in the various age groups. Subjects with sickle allele in the heterozygous state (10.1%, 60/594) were distributed in all age groups and 10.0% (6/60) subjects survived beyond 45 years of age indicating better survival in comparison to sickle cell anemia subjects. Eighteen subjects (3.03%, 18/594) were found to have homozygous sickle cell anemia. Majority of these subjects (66.7%, 12/18) were in the 1–15 years age group. Their proportion progressively decreased with increasing age of the population and none of the subjects were alive beyond >45 years of age. This finding highlights the facts that sickle cell anemia patients have increased mortality and very few of them survive beyond the fifth decade. The age-wise distribution for alpha thalassemia subjects (both heterozygous and homozygous state) was similar to that of sickle allele in the heterozygous state.

In a multicentric study of college students and pregnant women, the overall prevalence of *β* thalassemia trait was found to be 2.78% in India [35]. The data on prevalence of *β* thalassemia in different tribal populations in India is scarce. The prevalence of *β* thalassemia trait in the Adivasi of Gujarat ranged from 1.1% to 2.24% [31]. In a study on the health status of tribes of Odisha, the prevalence of thalassemia allele varied from 1.9% in Kharias to 8% in Santala [19]. In our study the prevalence of *β* thalassemia allele was 3.4% in tribes studied which is comparable to the earlier reports. Interestingly this allele was confined in the Kandha tribe only. The only mutation found in the *β* thalassemia subjects was IVS1-5 (G→C).

## 5. Conclusion

Inherited hemoglobin disorders like alpha thalassemia and sickle gene are highly prevalent in the tribes of western Odisha. The high prevalence of deletional alpha thalassemia and sickle gene (in heterozygous or homozygous state) in tribal population in this region is probably due to selection pressure of endemic malaria in this part of India. This population is ideally suited for studying the mechanism of interaction and pathophysiological basis of malaria protection by inherited hemoglobin disorders like alpha thalassemia and sickle gene.

## Supplementary Material

We undertook a study entitled “Influence of heterozygous and homozygous alpha thalassemia on the severity of *P. falciparum * malaria in India.” sponsored by Department of Science and Technology (DST), Government of India, New Delhi. This was a case control study undertaken at V. S. S. Medical College, Burla, Odisha, from 2010 to 2013. All the confirmed cases of Plasmodium falciparum infection with severe malaria admitted in Department of Medicine, V.S.S. Medical College, Burla were included (N=279). Cases were categorized in to three groups (Normal alpha globin genotype, *αα*/*αα*; heterozygous alpha thalassemia, -*α*/*αα*; and homozygous alpha thalassemia, -*α*/-*α*). These cases in the three groups were compared with age, sex and ethnicity matched healthy controls (N=271) (Table-1). The prevalence of both heterozygous and homozygous alpha thalassemia was significantly lower (p<0.05) in severe malaria cases (38.7%) than in healthy controls (48.0%), which indicated that the deletional alpha thalassemia has a protective effect against severe *P falciparum* malaria. The clinical profile of all patients showed that the patients with alpha thalassemia (both heterozygous and homozygous) had a significant protective effect from cerebral malaria, multi organ dysfunction, Jaundice, Acute renal failure, and death from patients with normal alpha globin genes (p<0.05 by Fisher's exact test) (Table-2).

## Figures and Tables

**Table 1:** Demographic features of study subjects with allelic frequency of different hemoglobinopathies.

	Sahara	Kandha (Kutia)	Oraon	Gond	Kuda (Mirdhas)
Number (%)	252 (42.4%)	178 (30.0%)	12 (2.0%)	58 (9.8%)	94 (15.8%)
District	Bargarh and Kalahandi	Kalahandi	Kalahandi	Bargarh	Bargarh

Age (Years)	21.6 ± 18.3	18.7 ± 18.0	20.02 ± 13.5	27.9 ± 21.7	22.5 ± 19.2
Sex					
Male	118	96	8	26	44
Female	134	82	4	32	50
*β* ^S^ allele frequency	0.08	0.07	0	0.17	0.05
*β* ^T^ allele frequency	0	0.03	0	0	0
*α* ^−3.7^ allele frequency	0.3	0.41	0.25	0.28	0.28
*α* ^−4.2^ allele frequency	0.06	0.03	0	0	0.04

*β* ^S^: sickle gene mutation; *β* ^T^: *β* thalassemia gene mutation.

**Table 2:** Prevalence of alpha thalassemia allele, sickle allele, and *β* thalassemia allele in different age groups in tribal population.

Age wise distribution of subjects (*n* = 594) *n* (%)	Sickle allele		*β* thalassemia allele *n* (%)	Alpha thalassemia alleles *n* (%)
	Heterozygous state *n* (%)	Homozygous state *n* (%)		
1–15 years 310 (52.2%)	22 (7.1)	12 (3.9)	14 (4.5)	148 (47.7)
>15–30 years 126 (21.2%)	22 (17.5)	4 (3.2)	4 (3.2)	62 (49.2)
>30–45 years 82 (13.8%)	10 (12.2)	2 (2.4)	2 (2.4)	52 (63.4)
>45 years 76 (12.8%)	6 (7.9)	0 (0.0)	0 (0.0)	40 (52.6)

Total	60 (10.1)	18 (3.03)	20 (3.37)	302 (50.8)
