# Supplementary material for: Prevalence of Deletional Alpha Thalassemia and Sickle Gene in a Tribal Dominated Malaria Endemic Area of Eastern India
Source: ISRN Hematol. 2014 Mar 11;2014:745245. doi: 10.1155/2014/745245 (PMC3967634; doi:10.1155/2014/745245)
Supplement: Supplementary file 1 — We undertook a study entitled “Influence of heterozygous and homozygous alpha thalassemia on the severity of P. falciparum malaria in India.” sponsored by Department of Science and Technology (DST), Government of India, New Delhi. This was a case control study undertaken at V. S. S. Medical College, Burla, Odisha, from 2010 to 2013. All the confirmed cases of Plasmodium falciparum infection with severe malaria admitted in Department of Medicine, V.S.S. Medical College, Burla were included (N=279). Cases were categorized in to three groups (Normal alpha globin genotype, αα/αα; heterozygous alpha thalassemia, -α/αα; and homozygous alpha thalassemia, -α/-α). These cases in the three groups were compared with age, sex and ethnicity matched healthy controls (N=271) (Table-1). The prevalence of both heterozygous and homozygous alpha thalassemia was significantly lower (p<0.05) in severe malaria cases (38.7%) than in healthy controls (48.0%), which indicated that the deletional alpha thalassemia has a protective effect against severe P falciparum malaria. The clinical profile of all patients showed that the patients with alpha thalassemia (both heterozygous and homozygous) had a significant protective effect from cerebral malaria, multi organ dysfunction, Jaundice, Acute renal failure, and death from patients with normal alpha globin genes (p<0.05 by Fisher's exact test) (Table-2). [file 745245.f1.pdf]

Table-1. Distribution of alpha thalassemia genotypes among the severe malaria cases and healthy control:

	Normal Alpha Globin Genotype αα/αα N(%)	Heterozygous Alpha thalassemia -α/αα N(%)	Homozygous Alpha thalassemia -α/-α N(%)	Total deletional Alpha thalassemia
CONTROL (N=271)	141(52.0)	85(31.4)	45(16.6)	130(48.0)
CASES (N=279)	171(61.3)	65(23.3)	43(15.4)	108(38.7)

Table-2. Clinical characteristics of severe malaria cases at the time of admission with respect to their alpha thalassemia status.

Clinical features of severe malaria cases.	Total no. of severe malaria cases N=279	Normal alpha globin genotype αα/αα N=171 (A)	Heterozygous Alpha thalassemia -α/αα N=65 (B)	Homozygous Alpha thalassemia -α/-α N=43 (C)	Total deletional Alpha thalassemia N=108 (D)=(B)+(C)	p value
Cerebral malaria	76(27.2)	57 (33.3)	12(18.5)	7(16.3)	19(17.6)	$p<0.05$
Multi organ dysfunction	42(15.05)	32(18.7)	6(9.23)	4(9.3)	10(9.3)	$p<0.05$
Anemia	45(16.13)	29(16.9)	10(15.4)	6(13.9)	16(14.8)	$p>0.05$
Jaundice	83(29.7)	61(35.7)	14(21.5)	8(18.6)	22(20.4)	$p<0.05$
Acute renal failure	89(31.9)	67(39.2)	12(18.5)	10(23.3)	22(20.4)	$p<0.001$
Fatal outcome	24(8.6)	19(11.1)	3(4.6)	1(2.3)	4(3.7)	$p<0.05$
